# Gas Chromatography Olfactometry (GC-O) for the (Semi)Quantitative Screening of Wine Aroma

**DOI:** 10.3390/foods9121892

**Published:** 2020-12-18

**Authors:** Arancha de-la-Fuente-Blanco, Vicente Ferreira

**Affiliations:** Laboratorio de Análisis del Aroma y Enología (LAAE), Department of Analytical Chemistry, Instituto Agroalimentario de Aragón (IA2) (UNIZAR-CITA), Universidad de Zaragoza, c/Pedro Cerbuna 12, 50009 Zaragoza, Spain; arandlfb@unizar.es

**Keywords:** odorants, odours, flavour, sensobolome, odour zones, retention index, gas chromatography-olfactometry

## Abstract

This review discusses the different approaches developed by researchers in the last 40 years for the qualitative and semi-quantitative screening of odorants, with special emphasis in wine aroma profiling. In the first part, the aims and possibilities of Gas chromatography-olfactometry (GC-O) as odour-screening and aroma profiling technique are discussed. The critical difference between approaches is whether the ranking of odorants is carried out on an extract containing all the odorants present in the product, or on an extract representative of the odorants contained in the vapour phases that cause the odour and flavour. While the second alternative is more direct and can be more efficient, it requires a good understanding of the factors affecting orthonasal olfaction, handling volatiles (purging, trapping, eluting, and separating) and about the sensory assessment of GC effluents. The review also includes an updated list compiling all the odorants detected in wine by GC-O, including retention indexes and odour descriptions with a general guideline for the identification of odorants.

## 1. Wine Aroma

Wine is a very special food product whose value is increasingly associated with the set of characteristics, both extrinsic and intrinsic, responsible for the pleasure associated with its consumption. Extrinsic elements such as connections with geography and history, brand image, or perception of exclusivity, amplify the pleasure associated with the purely sensory perceptions, which are the intrinsic and primary elements of wine quality [1]. Within these sensory perceptions, complexity and aromatic balance are two key elements [2,3]. It should be remarked that the most appreciated wines rarely have explicit and easy to define aromas, rather they have complex aromatic notes in which some fruit and freshness perception is essential, along with other spicy, woody or toasted notes, depending on the type of wine. Note that, especially for experts, the absence of aromatic defects or deviations is also always an essential element of quality [4].

The set of wine aromatic perceptions includes all the different odours perceived through the nose during wine consumption. These odours change with time due to the progressive evaporation of the most volatile compounds once the wine is poured in the glass [5,6], changing both orthonasal and retronasal perceptions. Behind those odours there are several dozens of wine odorants able to reach our olfactory epithelia during wine consumption. The set of perceived olfactory perceptions are the result of various processes of modulation and integration of the primary olfactory signals produced by each one of the odorants. In the in-mouth perceptions, integration includes stimuli from the senses of taste and touch. All these integration processes make it difficult to understand the relationship between the primary olfactory inputs and the perceived aroma. For instance, cooperative associations between very weak odorants of more or less similar odours can produce clear and net odours [7,8], or the strong suppression effects of some components such as 2,4,6-trichloroanisole (TCA) or higher alcohols [9] can completely suppress other relevant odours. The corollary is that understanding wine odour characteristics requires more than just studying its most intense odorants.

Some odorants are common to all wines and can be considered “constitutive” of wine. Among them, the secondary volatile metabolites of alcoholic fermentation, or in the case of oak aged wines, the wood extractable volatiles. Most of these “constitutive” volatiles are also relatively easy quantified by GC-MS since they are in affordable concentration ranges (several µg-mg/L). There is, however, a second group of relatively common odorants, many of which derive from the grape, which can be found in much wider concentration ranges. Terpenes, norisoprenoids, volatile phenols, vanillins, rotundone, methoxypyrazines or polyfunctional mercaptans are found in this group. Some of them are responsible for the specific aromatic properties of certain types of wine. The low concentrations at which they can become active can complicate the analytical control, particularly in the cases of polyfunctional mercaptans, methoxypyrazines or rotundone. Something similar happens to some potential off-odours, such as TCA and other halophenols, or 1-octen-3-one, *E*-2-nonenal and other fatty acid-derived odorants. The list of potentially relevant aroma compounds, both positive and negative, increases steadily with time. This is in part the logical consequence of our scientific and technical progress, but unfortunately, and particularly for negative aroma compounds, such increase is a side consequence of the increasingly frequent anomalous climatological phenomena affecting grape maturation.

In this quantitatively complex scenario, and despite the analytical power of current GC-MS and HPLC-MS instrumentation, we are going to be constantly compelled to evaluate both the presence of unexpected odorants in the aromatic profile, and to identify quantitative alterations of the aromatic profiles potentially responsible for odour imbalance. In both situations, GC-O can be very useful if its basics, potential and limitations are well understood.

## 2. Gas Chromatography-Olfactometry as a Technique for Screening Odour-Active Molecules

Gas chromatography-olfactometry (GC-O) has been used almost since the introduction of gas chromatography, as the human nose is the most appropriate detector to monitor the presence of an odorant in the effluent of a gas chromatograph [10]. For GC-O, the flow at the outlet of the chromatographic column is divided into two branches by means of a union or Y-joint, one that carries the analytes to an instrumental detector (FID, MS, …); and another one that takes them to an olfactometric port, where the human nose acts as a detector of great sensitivity and selectivity. The first forms of GC-O consisted simply on the sensory description of the effluent from the chromatographic column with the aim of assessing whether the chromatographic peak was odour active. In the case of grapes and wines, the first reports date from the 70′s, when the technique was first used to identify C6 alcohols and aldehydes as responsible of the leafy odour of grape leaves [11] and to monitor changes in aroma composition during aging [12]. One of its first successes was the identification of furaneol as key off-odour of the wines made with *V. labrusca* hybrids [13].

The potential of GC-O as a screening technique able to rank the odorants present in a product attending to their potential relevance in the product begun to be recognized in the 80′s with the pioneer works of Acree, et al. [14] and Schieberle and Grosch [15]. These authors introduced the two first systematic approaches for obtaining quantitative parameters related to the olfactory importance of an odorant in a given product: charm analysis and AEDA, respectively. Charm is the acronym for Combined Hedonic Aroma Response Measurements and AEDA for Aroma Extract Dilution Analysis. The techniques will be later presented and discussed with more detail. Now, some previous disquisitions about the goal of the GC-O screening operation will be elaborated to clarify some concepts which often are not correctly understood by researchers.

The obvious goal of the GC-O screening operation is to rank the odorants present in the product attending to their relative implication on the aroma-related sensory properties of the product. For this, the shortest way is to carry out the GC-O screening operation on an extract whose composition closely resembles those of the vapour phases emanating from the product during its olfaction and/or consumption. However, producing such an extract is not straightforward at present, and it was yet more complicated 30 years ago. By then, early researchers realized that the direct GC-O study of headspaces (usually carried out under equilibrium conditions) yielded just a very little fraction of the most volatile odorants present in the product, which at the end, resulted to be not really much important on its odour and flavour [16,17]. Those headspace fractions were also so diluted that identification was very difficult. Because of these reasons, most researchers decided to get a “total extract” from the product, and even today, the GC-O operation is most often carried out on such total extract after the corresponding operations of cleaning and concentration. A “total extract” can be easily obtained from any product. For that, the product just has to be extracted with relatively high volumes of a solvent of medium polarity (diethyl ether or dichloromethane), preferably using several consecutive extractions. This type of extracts can easily contain 100% of the odorants present in the original product, and from this point of view, they are “representative” of the product. However, it is of the outmost importance to understand that these types of extract cannot provide unbiased estimations of the relative importance of the different odorants in the sensory properties of the product. The reason for this has to do with the fact that in GC, all the volatile components introduced in the chromatographic column end volatilized and reach the detector, regardless of their volatility. On the contrary, in the original product the different odorants are transferred to the vapour phases at very different proportions, depending on their specific volatilities in the product matrix. These volatilities do not depend only on the size and boiling point of the odorant, but on the interactions that it establishes with the matrix. Unfortunately, these volatilities in aqueous matrixes can be so different between odorants that can completely invalidate the ranking obtained in the GC-O operation carried out on the total extract. To illustrate this situation let’s take as example two similarly powerful odorants with very different polarities: vanillin and 2,4,6-trichloroanisol (TCA), whose properties are summarized in Table 1.

As can be seen, both odorants have very similar odour thresholds in air, which indicates that they are similarly powerful, i.e., our noses require similar numbers of molecules of both components to elicit a detectable odour signal. However, their odour thresholds in water differ by more than 6 orders of magnitude. This difference is due to the different polarities of both molecules. While TCA is quite hydrophobic and scarcely soluble in water (log P = 4.1, Wsol = 10 mg L^−1^), vanillin is quite hydrophilic and water soluble (log P = 0.59, Wsol = 6.9 g L^−1^). The volatility from aqueous solutions, is given by the Henry’s volatility constant, and as can be seen, that of TCA is more than 5 orders of magnitude higher than that of vanillin, which basically tells us that TCA is more than 5 orders of magnitude more easily transferred from an aqueous solution to the vapour phase, which explains its much lower odour threshold in water. Let’s recall, however, what will be the outcome of a GC-O experiment carried out on a “total extract” obtained from an aqueous product in which both compounds are present at 1 µg L^−1^. As both components will be equally extracted, the GC-O operation will tell us that both odorants are equally important in the original product. The truth, however, is that TCA is 300 times above threshold, while vanillin is 100 times below. This example should let us conclude that any GC-O screening operation carried out on a “total extract” most likely provides a biased hierarchy of odorants. The odorants more retained (less volatile) in the original matrix will be highly over-estimated. In aqueous and hydroalcoholic matrixes, this will happen to all the polar and water-soluble odorants (acids, alcohols, phenols, mercaptans…).

Aware of this bias, the most widely used and accepted GC-O screening strategy, originally proposed by Schieberle and Grosch [15], also known as “sensomic” or “molecular science concept”, includes as part of the screening strategy the experimental determination of so-called odour activity values (OAVs, quotients concentration/odour threshold) of all the odorants identified in the GC-O screening. Once the concentration of the odorant is corrected by its odour threshold in the product matrix, the volatility differences responsible for the bias of the olfactometric screening become corrected, so that the OAV list provides an un-biased hierarchy of the odorants in the product, i.e., in this strategy the ranking provided by the GC-O screening is simply an intermediate operation whose goal is to identify the molecules with odour in the product but cannot anticipate their role on the sensory properties.

Experience has demonstrated that the “molecular science concept” works. However, it can be argued that it is time consuming and quite inefficient, since all odorants found in the total extract have to be identified and quantified, while only a little fraction are relevant. Any strategy providing extracts for GC-O representative not of the product, but of the vapour phases emanated from the product, should make it possible to make an earlier selection of the “a priori” most relevant odorants, saving much work. This requires to overcome the difficulties of obtaining headspace fractions fulfilling the following two requirements:

(1) To be concentrated enough to detect and identify all relevant odorant of the product
(2) To be truly representative of the vapour phases emanated from the product

These two conditions are nowadays affordable. The comparison between both philosophies, with some of their advantages and disadvantages are summarized in Table 2.

To the best of our knowledge, the above classification is proposed for the first time. In general, researchers tend to name and classify the GC-O screening operation attending to the specific olfactometric strategy followed (for instance AEDA, NIF, posterior intensity or Osme). However, the olfactometric strategy is secondary, since it rather affects to the how, while the key definitory parameter of the GC-O is its goal, which defines the what. Keeping in mind these two different possibilities, the two main elements of a GC-O screening operation, namely obtaining the extract and the GC-O strategy, will be briefly discussed.

## 3. Sample Preparation Strategies

### 3.1. Preparation of “Total Extracts”

Most researchers choose the classic option of making a total sample extract (complete aromatic sensobolome) [18,19,20], because of concerns about the representativity and concentration achieved by headspace extracts. The techniques that have been most used in the total extraction technique are liquid-liquid extraction (generally dichloromethane or ether/pentane) or solid phase extraction (SPE), which will be briefly commented.

Liquid-liquid extraction (LLE), also known as solvent extraction, is used to separate chemicals from one solution to another based on the different solubility of the analytes in two immiscible solvents. Dichloromethane (DCM) is the most widely used solvent to extract odorants due to its relatively high polarity (log P = 1.25), low solubility in water (17 g L^−1^) and from water (1.7 g L^−1^) (4 and 10 times less than ethyl ether), ease of evaporation (40 °C), low flammability, chemical stability and high purity. Its drawbacks are toxicity, high molecular weight and presence of Cl atoms. LLE most often uses large volumes of solvents, and consecutive extractions. In general, extraction is oversized to ensure total extraction (4–6 successive extractions). The so obtained liquid-extract will contain 100% of all volatiles in the original product, but surely will contain as well little to medium amounts of non-volatile material (waxes, chlorophylls, sterols, fats, fatty acids, and some not very polar polyphenols), little amounts of water, and in the case of wine, alcohol. It will be also too diluted and has to be concentrated by distillation of the solvent.

The water can be easily removed by drying the extract with anhydrous Na_2_SO_4_ (1 g/10 mL) overnight; ethanol can be also partially removed (if required) by washing the partially concentrated extract several times with brine (1:1 volume). Elimination of the non-volatile material can be trickier. Normal GC vaporizing injections can tolerate little amounts of non-volatile material in the extracts, but at some point, the non-volatile material in the GC inlets will inevitably introduce “activity” which will provoke peak distortion and with time, even the disappearance of active analytes, such as mercaptans, aldehydes or other polar compounds. Additionally, some artefacts, namely odorants produced by the thermal degradation of a non-volatile precursor in the injector, can be also observed. Therefore, dirty extracts will have to be compulsorily cleaned.

Because of that, researchers at Garching (Munich, Germany) developed a specific vacuum distillation technique known as solvent-assisted flavour evaporation (SAFE) [21] in which the extract is forced to evaporate under a strong vacuum, leaving behind the non-volatile material. Evaporated vapours are further condensed with the help of liquid nitrogen in a cleverly designed glass instrument. The SAFE-treated extract is completely free of non-volatile material and, well worked out, has an odorant composition very close to the original extract. This extract can then be further concentrated by careful evaporation (by micro-distillation preferably) to the level required. Concentration factors well above 1000 with respect to the original sample can be easily attained.

A second possibility for getting total extracts from liquid matrixes, such as wine, is solid phase extraction (SPE). For getting full advantage of this technique “new” generation polymeric sorbents with high active surface areas and eventually polar functionalities should be used. These sorbents can provide distribution coefficients 1000 times above those obtained with dichloromethane, 100 times better than those obtained with C18 sorbents and more than 20 times better than those obtained with classical styrene-divinylbenzene copolymers, such as XAD-2 or XAD-4, as is demonstrated in different references [22,23,24]. In a comparative study, the highest distribution coefficients from wine and hydro-alcoholic solutions were obtained with LiChrolut EN from Merck (Darmstadt, Germany) and Isolute ENV+ from Biotage (Uppsala, Sweden) [24]. For these two sorbents, the geometric mean of the liquid-solid distribution coefficients of different odorants between hydro-alcoholic solution and the resins is around 10,000.

The advantage of SPE extraction is that the process is in fact a chromatographic separation, carried out in the so-called frontal development mode, in which the sample is being continuously fed to the SPE bed. The analytes within the bed travel at different rates, depending on their liquid-solid distribution coefficients. A distribution coefficient 10,000 implies that such analyte will travel 10,000 times slower than the sample solvent, assuming within the SPE bed a 1:1 phase ratio. Then, if a 10 mm bed, with an approximated internal liquid volume (dead volume) of 0.2 mL, is percolated with 200 mL of sample, such analyte will have advanced just 1 mm within the chromatographic bed. A second analyte with a distribution coefficient 1000, will have advanced 10 mm so that will begin to become lost. But if the sample applied is limited to 100 mL, then this second analyte will occupy just the 5 first mm of the bed [23]. For getting quantitative extracts, the amount of sample applied per volume of SPE bed is determined by the most polar and worst extractable wine odorants. In wine, these are polar compounds such as furaneol, sotolon or vanillin. For getting completely quantitative recoveries of these compounds, the SPE bed should contain around 15 mg or sorbent per mL of wine processed [25], but ratios of 4 mg or sorbent per mL of wine still makes it possible to obtain very good extracts [26]. Complete elution with dichloromethane or dichloromethane with 5% methanol requires just 7.5 µL of solvent per mg of bed. Attending to this, 100 mL of wine can be extracted with just a 400 mg SPE bed, including a small washing up with 4 mL of a 13% ethanol: water mixture and the aroma will be recovered with just 3 mL of solvent, after drying the SPE bed. This extract is cleaner than those obtained by direct extraction and it can be, in general, safely concentrated to 0.3 or 0.1 mL (achieving concentration factors between 300 and 1000) and injected in the GC inlets, provided that the inserts contain some inert filling, such as silanized wool, and that the column is conveniently protected by a guard column.

In spite of the strong simplification in sample workup introduced by SPE, we gave up with total extracts for GC-O screening of wine more than 15 years ago. Apart from the strong overvaluation of polar odorants, as discussed in the previous section, the extracts contain so many odorants that many odour zones in the olfactogram overlap, making difficult the assessment and the identification of the odorant [27,28]. However, some other researchers value the advantages of this strategy and have used it recently [29,30].

### 3.2. Preparation of Headspace-Extracts

As odorants are volatile compounds, headspace approaches have up to three initial important advantages over strategies based on the direct extraction of the odorants. First, these extracts have a much smaller risk of overvaluing odorants poorly transferred to the headspace; second, extracts are completely free of non-volatile material, and third; depending on the approach, most volatile odorants won’t be lost by evaporation or hidden by the solvent. However, because of a number of reasons, the study of headspace is more complex of what it may seem. Consequently, we will first describe and comment the different technical possibilities and will secondly discuss about the question of representativity.

There are three different major approaches to the study of headspace:

(1) Direct sampling of equilibrated headspace (static headspace)
(2) SPME (or equivalent) sampling of headspace
(3) Dynamic sampling of headspace (purge and trap)

Static headspace techniques are convenient for the analytical determination of a number of volatile compounds but have a major limitation for being useful for GC-O screening: their low sensitivity. Consider that in a simple inhalation we can uptake up to 700 mL of air, while static headspace techniques can hardly deal with 4 or 5 mL of headspace. These techniques, in which the equilibrated headspace is directly transferred to the GC-O system, are limited by the small flows used in the GC-capillary columns. With flows in the mL/min range, introducing 5 mL takes at least 5 min and produces extremely broad peaks for the most volatile compounds, which usually are poorly retained at the minimum temperature of the GC. Peak broadening can be overcome by using a cryofocussing unit, but yet, the maximum volume injected will be limited to some few mL. Higher volumes will introduce into the cryofocusing unit enough vapours of water and ethanol to produce crystals (ice plugs or ethanol-ice plugs) which will ruin the chromatographic separation.

These difficulties are easily overcome by sampling the headspace with a SPME fibre. The SPME fibres have a very low affinity for water and ethanol and can provide huge concentration factors for many volatile compounds. This technique, introduced by Pawliszyn in the 90s [31], has an amazing ability to concentrate volatile compounds and yet it can be easily used with simple GC inlets, not requiring special equipment. These superior advantages make it extremely easy to get very good chromatograms, and very low detection limits, which has boosted its popularity between researchers. In fact, a simple search in The Web of Knowledge with the words SPME and wine, yields more than 1100 hits. The technique has been also widely applied as the sampling system for olfactometric analysis in wines and other alcoholic beverages so that making a comprehensive analysis is out of the scope of the present review. We will try, instead, to delimit the strengths and weaknesses of the technique for the purpose of GC-O profiling of wine.

A main characteristic of SPME is the low volume of extracting phase, which can be as low as 0.1 µL and is never higher than 1.5 µL. Is this little amount of phase what makes possible a fast desorption in a simple GC split/splitless injector (with an adequate liner). Very narrow peaks, even for the most volatile compounds, are obtained as a result. A second advantage of the little amount of phase is its potential to concentrate analytes. If all the molecules of an odorant are satisfactorily extracted from a 10 mL sample volume by the SPME fibre, the injection would provide a 10,000-concentration factor in a single operation. With standard MS detection this would suffice to measure molecules at ppt level. These benefits are so appealing that have seduced many researchers. However, little amounts of phase have two negative side effects that make the approach unreliable in wine GC-O: saturation and diversity in recoveries.

Regarding saturation, a partition phase (such as PDMS or carbowax) can absorb 10% of its weight, so that, the maximum mass of volatiles that a SPME fibre can hold is between 0.01 and 0.15 mg. However, a 10 mL volume of wine contains more than 3 mg of volatiles, excluding ethanol, acetic acid and ethyl acetate. This implies that the SPME fibre will become saturated whenever contacts volumes of wine higher than 0.02–0.5 mL, depending on the type of fibre. Under saturation, extraction will be highly sample and procedure-specific. The relative composition of the extract will depend on hardly controlled factors, such as the ethanol level, or the sample content in major volatiles (ethyl acetate, acetic acid, higher alcohols…) [32]. Saturation problems will be still stronger if the fibre contains adsorbents, such as carboxen. Matrix effects in quantitative analysis can be partly compensated by the use of adequate internal standards, but this can hardly be done in GC-O.

Secondly, recoveries will be highly variable between the different odorants. The odorants more favoured will be those ones with high logP (scarcely soluble in wine) of sizes around 120–220 g/Mol (volatile enough to be transferred to the headspace, and heavy enough to be stabilized in the fibre). Examples of odorants of this type are the ethyl esters of hexanoic, octanoic, decanoic or of isovaleric acid or TCA. Recoveries in the SPME operation for these compounds can be between 10 and 50%. By contrast, least favoured odorants will be those of high polarity (low logP), such as furaneol or vanillin. As these are highly solubilized in wine, they will be transferred to the fibre very slowly and always in very low proportions. Recoveries for these compounds can be below 0.001%. Consequently, the GC-O screening operation will overvalue the least soluble (and most volatile) odorants and will undervalue those least volatile in the original matrix. It can be reasonably argued that this is what happens in normal olfaction, but not with differences of the magnitude observed in headspace SPME.

Note that all these problems can go easily undetected, since the chromatograms can look perfect and the process be repetitive.

We can therefore conclude, that normal SPME is, by its intrinsic limitations (which are also its strengths), not well suited to provide an objective hierarchy of the odorants in wine by GC-O screening, nor adequate to provide fine comparisons between GC-O profiles of very different wines. This does not mean that it cannot be useful to detect and identify specific odorants, such as off-odours or some target aroma impact compounds, or that can be a very good complement for the study of the most volatile odorants of wine, as the extended literature on the topic demonstrates [33,34].

Some of these limitations can be partly overcome with the improved versions of SPME devices, such as the SPME-arrow, which have come into scene just after the expiration of the limitations imposed by the patent of SPME [35]. These systems keep the simplicity of the SPME design, and its adaptation to routine work requires significant but not major changes in the GC system. The major advantage derives from their higher masses of sorbent (6–20 times higher) and higher exposed surfaces (5–7 times larger). This will limit the effects linked to the mass-saturation of the fibre (matrix effects), and will provide higher recoveries for all compounds, particularly to those poorly transferred to the headspace, which suggests that these systems have an interesting potential for GC-O screening of wine.

Finally, purge and trap strategies are dynamic strategies in which, with the help of an external stream of inert gas, volatiles emanated or dragged from the wine are trapped in an adsorbent bed. After the trapping period, trapped volatiles are desorbed, well by elution with a solvent, well by direct thermal desorption. Only in the first case there is a physical extract with which the GC-O screening operation will be carried out. In the second case, except with highly advanced thermal desorption units, the purge and trap process will have to be repeated as many times as required to carry out the GC-O process. The variables defining the purge and trap process are those controlling the release of the volatiles from the sample and those others referred to the trapping and desorption.

From the point of view of the release of volatiles, the purge and trap system has to simulate as much as possible orthonasal olfaction. Olfaction, as when smelling a glass of wine, is basically a dilution process in which a simple sniff takes 700 mL of headspace at 100 mL s^−1^ [36,37]. The volume of headspace sampled is so large, and it is taken so fast, that even agitating the wine in the glass, the process becomes limited by the transference of volatiles from the liquid to the gas phase. This is a mass-transfer controlled process, in which the composition of the vapour phase is very different to those found in equilibrium [38]. It has been demonstrated that anything facilitating the transfer of volatiles from the liquid to the gas phase, such as bubbling, shaking, increased evaporation surfaces, warming up, etc., provide extracts closer to equilibrium conditions [39] in which, in comparison to real olfaction conditions, the most volatile odorants are over-represented versus the least volatile. Therefore, is essential to avoid agitation, bubbling or heating, and relatively large streams of gas have to be used. The path from the liquid to the sorbent has also to be neat and short [38,39].

Regarding the trapping of volatiles, Tenax-TA has become a sort of standard for systems using thermal desorption, because of the high temperatures at which it can be used. However, its sorption capacity is quite limited in comparison to divinylbenzene-polystyrene copolymers, such as Bond Elute ENV or LiChrolut EN, providing breakthrough volumes 2 to 3 orders of magnitude smaller than these other sorbents [38,40]. In addition, the adsorption capabilities of Tenax are particularly limited in the presence of ethanol. Therefore, unless thermal desorption is strictly required, other polymeric sorbents are a much better choice. For elution with a solvent, it has been demonstrated that best results are obtained using dichloromethane containing a 5% in methanol [38]. The presence of methanol is required to improve the elution of polar odorants, such as furaneol and sotolon.

Balancing advantages and disadvantages of headspace strategies in comparison to total-extraction strategies, it should be considered that headspace strategies require a far more delicate chromatographic work. Headspace extracts, by nature, will be less concentrated, particularly in polar odorants highly retained in the wine matrix. These polar odorants, when present at very low concentrations, will be very sensitive to any kind of adsorptive activity in the chromatographic inlets, including injector, pre-column, column and detector. The chromatographic system has consequently to be continuously and thoroughly checked [41] to ensure that the level of activity does not affect to the chemically most active odorants, such as mercaptans, amines, aldehydes or highly polar molecules. Activity will not only distort the chromatographic peaks, but odorants present at low concentrations can be completely lost by adsorption in active sites. The inconclusive results reported by some authors about the relative efficiency of GC-O systems [42], may be related to activity problems, as the suspiciously low number of polar odorants detected suggests.

## 4. Olfactometric Strategies

GC-O was initially devised simply as an auxiliary technique to help in the identification of odorants. However, in the late 1980s various researchers begun conceiving it as a bioassay able to provide an estimation of the relative important of the odorants present in the same product. As human assessors can perform different tasks with the smells eluting out of the column (detecting, measuring duration, measuring intensity, assessing qualities) different strategies have been proposed for semiquantitative GC-O screening. Those strategies can be classified into three major types:

(1) Based on the determination of thresholds (AEDA, Charm analysis)
(2) Based on the distribution of thresholds among judges (detection frequency) (NIF, SNIF)
(3) Based on the measurement of odour intensity (Posterior Intensity, OSME, Finger Span)

As these techniques have been already the subject of review [43,44], here they will be just briefly commented.

### 4.1. Strategies Based on Determination of Thresholds

Two techniques have been developed within this category, AEDA (for Aroma Extract Dilution Analysis, developed in 1987 by Schieberle and Grosch [15]) and CHARM (Combined Hedonic Aroma Response Measurements, proposed by Acree, et al. [14] in 1984). Both techniques are based on the sequential dilution of the aroma extract (following a factor R, where R is usually between 2 and 5).

In AEDA, dilutions are presented sequentially and are often smelled just by one or two judges, usually the researchers carrying out the study. In this technique, each detected odorant is assigned a dilution factor (FD), which corresponds to the last dilution at which the odorant was detected at least by one of the judges. The representation of the FDs vs. the retention indexes is called “aromagram”. CHARM analysis has a more refined setup. Dilutions are presented in a random order and the signal is created with the help of a computer. The judges participating in the study press a key of the keyboard during the duration of an odour, so that by combining (averaging) the signals of the different judges at the different dilutions, a “peak” (charm peak) for each detected odorant is formed. The signal is the area of the peak (charm area). The height of the charm peak is coincident with the FD measured in AEDA.

### 4.2. Strategies Based on the Distribution of Odour Thresholds among Judges (Detection Frequency)

These strategies make use of the differences in thresholds between individuals. For standard aroma compounds thresholds between the more sensitive 5% of the population and the 5% least sensitive differ by 1 to 2 orders of magnitude [45]. This implies that any odorant in the extract at concentrations within the ranges 0.1 * C_Threshold_–10 * C_Threshold_ will be detected just by a fraction of the members of a sensory panel. The fraction detecting the odorant will increase with the concentration following a logistic regression line. This property can be satisfactorily exploited to rank the odorants present in the effluent attending to their concentrations in the extract relative to their corresponding thresholds.

Nasal impact frequency (NIF) and surface of nasal impact frequency (SNIF) are the two variants of methods based on measurement of the frequency. The NIF technique was proposed by Pollien, et al. [46] and Van Ruth and Roozen [47] in the 1990s and assigns to each detected odorant a number between 0 and 100% corresponding to the proportion of judges detecting the odorant (NIF value). In the SNIF technique the judges also measure the duration of the odours. The signal is the mean duration multiplied by the frequency of detection and it is called SNIF [48]. These strategies have been demonstrated a perfect quantitative performance [49] but require a relatively large sensory panel and have a limited dynamic range.

### 4.3. Strategies Based on the Measurement of Odour Intensity

These strategies make use of a sensory panel trained for measuring the intensity of the odours eluting out of the GC-O system. There are two basic variants: time-intensity methods, such as OSME, and posterior-intensity methods, such as Finger Span or Frequency × Intensity methods.

In time-intensity methods, such as the OSME, the panellists are instructed to make a continuous assessment of the odour intensity of the effluent, somehow imitating a chromatographic detector. For that, the panellists move an actuator connected to a potentiometer, through a 15 cm path [50]. A verbal description of the odour is also recorded. The subjects perform several repetitions of each analysis and with the average of the intensities obtained for an odorant, the aromatic profile of the extract, the “osmogram”, is obtained. The output looks much like a FID chromatogram. The technique, however, is poorly repetitive, which can be attributed to the difficulty of the sensory task assigned to the judges.

Better results are obtained by methods treating each one of the odours detected in the effluent as independent simple events, assigning to each of them an intensity value, regardless of their duration. This sensory task is better suited to our sensory abilities, and after some training, fairly repetitive results can be obtained. Two different possibilities have been proposed: the Finger Span method and direct intensity measurement methods with or without measuring frequency.

The Finger Span method was proposed by Etiévant, et al. [51] in 1999, and takes advantage of the demonstrated relationship between the intensity of a sensory stimulus and some cross modal responses, such as the degree of opening between the thumb and index fingers or “finger span” [52,53]. In the proposed method, the index and thumb fingers of the judge are connected to a resistance that records when and how much the gap between the fingers is opened in response to the intensity of the perceived odorant [53]. The description of the quality of the odour detected is also recorded with a voice recording system.

Other researchers make use of a global estimation of the intensity of the odour (magnitude estimation) using a simple scale, combined or not with the measurement of frequency of detection. Given the difficulty of the task, the odour scale used is deliberately simple. Typically, a 7 point scale (0–3 with half values allowed) is used and combined with the measurement of frequency [54] as previously proposed by Dravnieks [55]. The quantitative performance of the technique was demonstrated by Ferreira, et al. [56].

### 4.4. Choosing the Most Adequate Olfactometric Strategy

A corollary of the previous discussion about the type of extract on which the GC-O is carried out, is that if we have chosen a total extract, the hierarchy of odorants provided by the GC-O will be poorly correlated to the real importance of the odorants in the original matrix. If this is the case, and we already have assumed that we will identify, quantify and normalize by the corresponding threshold all the odorants detected in the operation, then there is not much demand on the GC-O operation, and whatever strategy will work. This is the reason why AEDA, because of its simplicity, and in spite of their drawbacks as it is usually practiced [57], is often the technique of choice.

On the contrary, an extract truly representative of the vapour phases of the product deserves a fine sensory work to obtain a good ranking of odorants. This implies using a sensory panel to account for the differences between individuals. AEDA or Charm analysis using a smaller number of more separated dilutions with more assessors can be considered [57,58]. Such approaches will provide a reliable rank of odorants attending to their relative potencies (ratios Concentration/Threshold) in the sample. However, as the relationships Intensity/Concentration for different odorants follow power laws with different exponents, such ranking may not perfectly reflect the rank of odorants by intensity [59,60], which is the one majorly determining the odour properties of the product. NIF and SNIF techniques have the advantage that assessors do not require much training. Nevertheless, these techniques have a limited dynamic range and can hardly classify the odorants into 4 or 5 different categories, having particular difficulty to rank the most intense odorants [61]. On its side, intensity measurement techniques require training the sensory panel, but they can provide a ranking of the odorants based on relative intensities, which a priori, should be closely related to the aroma properties of the product. The usefulness of this approach for the identification of the odorants responsible for sensory differences between different wines [62,63], subtle differences between similar wines [64,65], defects [66] or even for modelling wine quality [67] has been repeatedly demonstrated.

## 5. Identification the Odorants Detected in Wine by GC-O

The GC-O process (sample extraction, concentration and GC-O) provides as output a hierarchical list with the different odours detected in the experiment, ordered according to the parameter measured by the chosen GC-O strategy, together with the retention time (possibly in a single GC column) and its sensory descriptor. Additional steps required to provide a reasonable proposal for the identities of the detected odorants include standardization of retention times, recording them in a second GC column and confirmation of the identity of the chemical [68].

To standardize retention times, a solution containing n-alkanes with between 6 and 24 carbon atoms must be injected in the GC-O system under the same conditions used for the samples. Retention times are then transformed into retention indexes (RI) using the formula:

(1) $$RI=100*\left[\frac{t_{Ri}-t_{Rz}}{t_{R\left(z+1\right)}-t_{Rz}}+z\right]$$

where $t_{Ri}$ is the retention time of the odour zone *i*; $t_{Rz}$ and $t_{R\left(z+1\right)}$ are the retention times of the n-alkanes eluting immediately before (*z*) and after (*z* + 1) the odour zone; and *z* is the number of carbon atoms in such *n*-alkane. For odour zones eluting close to some of the major volatiles in the chromatogram, such as isoamyl alcohol, the retention indexes estimated with alkanes injected separately will not be accurate. In these cases, best results will be obtained by direct addition of the couple of alkanes bracketing the odour zone to the sample extract. In dilution techniques, such as AEDA, retention times of the odour zones taken at highest dilutions will be more accurate.

The RI in a single GC-O system is not enough to propose a likely candidate for a given odour, except in obvious cases, such as those of acetic acid, isoamyl alcohol or β-phenylethanol. It is compulsory to have experimental RIs in a second chromatographic column with a stationary phase of different polarity. Most typically, the main experiment is carried out in column with a Carbowax 20M phase (DB-Wax or equivalent), which can provide perfectly symmetrical peaks for alcohols and acids, major compounds in all fermented products. The complementary phase is most usually a dimethylpolysiloxane containing a 5% of phenyl substituents (DB-5 or equivalent). Most chromatography suppliers offer high quality versions of these two phases with minimum activity towards active molecules, making it possible to get Gaussian peaks even for the most difficult odorants, such as methional, phenylacetaldehyde, 3-mercaptohexanol or furaneol. As previously mentioned, the inertness of the chromatographic system has to be frequently verified through the injection of activity-test mixtures [41,69]. In GC-O experiments carried out with highly concentrated extracts, such as those obtained by total extraction, the number of odorants can be too high for a reliable association of the odour zones detected in the two columns. In those cases, it can be necessary to use a GC-O-GC-O system with the two main chromatographic columns in tandem, so that unclearly assigned odour zones can be specifically transferred from the main column to the second. Alternatively, a 4-port valve can be installed in the GC-O system to trap conflicting areas and transfer them to the second column [70]. A simple device recently offered by Gerstel [71] makes the trick. Once we have a couple of reliable RIs for our odour zone, its provisional identity can be assigned with the help of Table 3, which provides an updated compilation of the different odorants detected in the different wine GC-O experiments carried out by different researchers. The table has 198 entries ranked attending to the experimental RI in a DB-Wax column obtained in our laboratory and contains 193 identified odorants and 5 unidentified odour zones. For 132 cases the experimental RI in the DB-5 column are also reported. Additional open databases such as those from Pubchem [72], NIST, Pherobase [73] or Flavornet [74] can be also consulted. However, as these databases compile data from many different sources higher uncertainties should be expected.

The identity of the compound has to be further confirmed by GC-MS. This should be done by injecting the extract in a GC-MS system equipped with a column similar to that used in the main GC-O in scan mode, and carefully measuring IRs with alkanes, best bracketing the targeted zones. The identification will be considered definitive when the pure standard is available and IRs in both GC columns, odours and mass spectral data coincide with those found experimentally. If the standard is not available, and the identification has to rely only in data reported in the literature, then the GC-MS data in the second column will be additionally required, and care will have to be taken regarding that the literature references that support the identification are truly backed in the use of synthesized reference compounds. There are several relevant reports with specific guidelines for the unequivocal identification of compounds in food chemistry [75,76].

In spite of the sensitivity of the most powerful GC-MS systems, some components may not be in sufficient concentration to generate a detectable mass spectrum in the headspace extracts. In this case, specific isolation strategies providing higher concentration and isolation levels should be carried out. Classical fractionation of highly concentrated extracts, such as those obtained in total extraction, on silica gel by flash chromatography or by semi-preparative HPLC should be considered.

## 6. Conclusions

This review highlights the different techniques used in the GC-O screening of any product, with a special emphasis on wine aroma profiling. It is argued that headspace-based GC-O screening techniques, carried out on orthonasal-like extracts on highly deactivated GC chromatographic systems, using trained sensory panels, are the most direct and powerful way to profile and control even subtle wine aroma changes.

## Figures and Tables

**Table 1:** Basic chemophysical properties and odour thresholds of vanillin and 2,4,6-trichloroanisol (TCA), two powerful odorants of very different polarities.

Property	Vanillin	2,4,6-Trichloroanisole (TCA)
Molecular weight (g mol^−1^)	152.2	211.5
Boiling point (°C)	285	241
Log P	0.59	4.11
Water solubility (mg L^−1^)	6875	10
Henry’s volatility constant (atm L mol^−1^ at 25 °C)	2.5 × 10^−9^	1.3 × 10^−4^
Log Koa	8.3	6.4
Odour threshold in air (µg L^−1^)	0.008	0.004
Odour threshold in water (µg L^−1^)	100	0.00003

**Table 2:** The two different general approaches to GC-O screening operation.

Questions	Total-Extract Based	Representative Headspace-Extract Based
Goal. What do we rank in the GC-O screening operation?	All the odorants present in the product, regardless of differences in transference rates to vapour phases	The odorants responsible for the odours and flavours elicited by the product
Emphasis	The odorants in the product	The odorants in the vapour phases emanated from the product
Extract. What should it contain?	All the odorants present in the product (at 100%)	The odorants present in the vapour phases emanated from the product
Result. What have we ranked?	Odorants attending to their olfactory importance in the extract	Odorants attending to their olfactory importance in the vapour phases
How results of the GC-O relate to the aroma-related sensory properties of the product?	Poorly. Olfactometric scores overemphasize the importance of the odorants more retained in the food matrix. A valid hierarchy is obtained only after OAV determination	If the extract is really representative of product headspaces, olfactometric scores should be closely related to aroma-related sensory properties of the product
Disadvantages/difficulties	Too much work. The hierarchy only will emerge after all OAVs have been estimated (all odorants have to be identified and quantified)	It is difficult to ensure that the extract is really representative of the vapor phases. Some odorants can be at too low levels in the extract for identification and quantification (a more concentrated extract may be necessary)
Global assessment	Excruciatingly long but trustful	Economical and efficient if a good and representative headspace extract is obtained

**Table 3:** Odour zones detected in wine extracts in a GC-O system in two chromatographic columns.

IR DBWax	IR DB5	Compound	Main Aromas
910		2-Methybutyraldehyde	Bread crust, closed
930		Pentanal	Aldehyde
934	730	Acetal (1,1-diethoxyethane)	Sweet, strawberry, aniseed
935		Propyl acetate	Alcoholic, sweet, fruit
937	755	Ethyl propanoate	Solvent, sweet, alcoholic
953	752	Ethyl isobutyrate (ethyl 2-methylpropanoate)	Lactic, strawberry, sweet
958		Diacetyl (2,3-butandione)	Butter, lactic
964		2,4,5-Trimethyl-1,3-dioxolane	Solvent, sweet
974		Isopropyl acetate	Fruit
995		Unknown	Plastic, adhesive
1005	833	Methyl 2-methylbutyrate	Fruit, sweet
1012	906	Unknown	Alcoholic, solvent, orange peel
1013	837	Isobutyl acetate	Fruit, apple
1020	943	α-Pinene	Mango, tropical, green, citrus
1025	803	Methyl 3-methylbutyrate	Fruit, sweet, anise
1037	801	Ethyl butyrate	Strawberry, sweet, lactic, fruit
1052	847	Ethyl 2-methylbutyrate	Fruit, sweet, strawberry, anise
1057	695	2,3-Pentanedione	Butter, cream
1057	852	2-Ethoxy-3,5-hexadiene	Geranium, metallic
1060		Dimethyl disulphide	Garlic, sweet, sulphur
1070	853	Ethyl 3-methylbutyrate (ethyl isovalerate)	Fruit, sweet, anise
1091	800	Hexanal	Herbaceous
1095		2,5-Dimethyl-1,4-dioxane	Green, grass
1098		Butyl acetate	Green, herbaceous
1102		Isobutanol (2-methylpropanol)	Fusel, humidity, bitter
1106		1-Hexen-3-one	Almond, toasted
1112	904	3-Methyl-2-buten-thiol	Rubber, hop
1127	875	Isoamyl acetate	Banana, adhesive
1135		4-Methyl-3-penten-2-one	Floral, green
1141	683	1-Penten-3-ol	Green, toasted
1142	941	Ethyl 2-methylpentanoate	Strawberry, fruit
1147	800	(*Z*)-3-hexenal	Green, grass
1150	1017	β-Pinene	Green, grass, green apple
1171	1032	3-Carene	Green, vegetal, grass, geranium
1185	960	Ethyl 3-methylpentanoate	Fruit, strawberry
1198	969	Ethyl 4-methylpentanoate	Fruit, anise
1200	857	(*E*)-2-Hexenal	Toasted
1218	753	Isoamyl alcohol	Foot odour, solvent, sharp
1223		1-Hepten-3-one	Mushroom
1245	996	Ethyl hexanoate	Anise, fruit, ester
1249		Unknown	Green, floral
1250		(*E*)-2-Heptenal	Green
1260		Acetoin	Lactic, fatty
1286	1014	Hexyl acetate	Banana
1292	952	Furfuryl ethyl ether	Solvent (Reflex)
1293	1045	Octanal	Citrus, rancid
1305	975	1-Octen-3-one	Mushroom
1310		2-Octanone	Rancid
1310		Unknown	Ester, grass
1315	860	2-Methyl-3-furanthiol	Fried, toasted
1320		2,5-Dimethylpyrazine	Spicy
1348	914	2,6-Dimethylpyrazine	Popcorn
1349	987	(*Z*)-2-Heptenal	Fried, rancid
1354	1126	*cis*-Rose oxide	Floral, rose, citrus
1358		Ethyl lactate	Synthetic, sharp
1366	872	1-Hexanol	Green, leaf, solvent
1378	986	(*Z*)-1,5-Octadien-3-one	Geranium, metallic
1378	1064	Unknown	Geranium, green
1380	985	Dimethyl trisulphide	Rubbish
1383		4-Mercapto-4-methyl-2-pentanone	Boxwood, green, urine
1395	848	(*Z*)-3-Hexen-1-ol	Green grass, grass
1399	1026	2,4,5-Trimethylthiazole	Geranium
1403	1114	Nonanal	Aldehyde, soap
1410	1080	1-Nonen-3-one	Mushroom
1416		Unknown	Strawberry
1425	1142	Ethyl cyclohexanoate	Anise, fruit, sweet, ester
1428	987	Ethyl 2-hydroxy-3-methylbutyrate	Strawberry
1429		2,3,5-Trimethylpyrazine	Earthy
1430	1201	Ethyl octanoate	Fruit, ester, sweet
1431	1015	2-Octanol	Rubbish
1433	1012	1-Octen-3-ol	Dust, toasted, citrus, mushroom
1434		Dimethyl methoxypyrazine	Cork, humidity
1436	907	Furfurylthiol	Toasted, coffee
1440	1080	(*E*)-2-Octenal	Citrus, bitter
1446	1094	2-Isopropyl-3-methoxypyrazine	Pepper, earthy, green
1453	909	Methional	Boiled vegetables
1455		Acetic acid	Vinegar
1460		Ethyl 2-hydroxy-3,3-dimethyl butyrate	Strawberry, fruit
1464	1070	Linalool oxide	Citrus, floral
1469	828	Furfural	Sweet wood, nut
1470		2,3-Diethyl-5-methylpyrazine	Meat
1473	1086	2-Ethyl-3, 5(or 6)-dimethylpyrazine	Green
1483		Citronellal	Lemon
1485		Copaene	Sweet wood
1488		Unknown	Rubber, new plastic
1489	1003	(*E, E*)-2,4-Heptadienal	Rancid, cucumber
1490	1209	Decanal	Floral, soap
1495		Propionic acid	Vinegar, boiled potato
1502	1159	Citronellal	Lemon, citrus, floral, mop
1507	1156	(*Z*)-2-Nonenal	Chlorine, rancid, aldehyde
1511	1145	3-Nonen-2-one	Rancid, wet, fried potato
1514	1172	3-sec-Butyl-2-methoxypyrazine	Pepper, earthy
1515	1156	2,3-Diethyl-5-methylpyrazine	Rubbish, rotten
1516		4-Vinylpyridine	Synthetic
1531	1060	Ethyl 2-hydroxy-4-methylpentanoate	Strawberry, ester
1533	1181	3-Isobutyl-2-methoxypyrazine	Pepper green
1533		Camphor	Mint, green
1538	1167	(*E*)-2-Nonenal	Melon, paper
1542		Unknown	Sweet, medicinal
1543	1315	Vitispirane	Floral, fruity
1556		Ethyl 3-hydroxybutyrate	Sweet
1561	796	2-Methylpropanoic acid (isobutyric acid)	Cheese
1562	1100	(*R/S*)-Linalool	Floral, citrus, muscatel
1569		Linalool acetate	Floral, fruity
1571	1154	(*E, Z*)-2,6-Nonadienal	Green, cucumber
1593	1158	2-Methylisoborneol	Mould, wet land
1618	1270	(*Z*)-2-Decenal	Chlorine, meat
1622	1021	2-Acetylpyrazine	Toasted, burned, coffee
1642	822	Butyric acid	Vomit, cheese
1655	1106	2 (3 or 4)-Methylbenzaldehyde	Burnt hair
1656	1080	Benzene methanethiol	Burnt hair
1660	1050	Phenylacetaldehyde	Floral, green
1662		2-Acetylthiazole	Roasted bread
1666		Undecenal	Aldehyde
1670		2-Methyl-3-(methyldithio)furan	Fried, barbecue
1674	1261	(*E*)-2-Decenal	Green, rancid
1680	878	3-Methylbutyric acid (isovaleric acid)	Foot odour, cheese, perspiration
1717	1217	(*E, E*)-2,4-Nonadienal	Rancid, toasted
1717		(-)-Borneol	Earth, mould
1717		2-Phenylethanethiol	Sulphur, plastic
1723	910	Methionol (3-methylthiopropanol)	Boiled potato, rubber, plastic
1725		Decadienal	Aldehyde
1732	1254	3-Mercaptohexyl acetate	Boxwood, basil
1735	1195	α-Terpineol	Anise, green
1738	1430	Dodecanal	Metallic, sea
1740		3-Methyl-2,4-nonanedione	Honey, strong
1748	1162	Borneol	Camphor, anise
1753	1149	Benzyl acetate	Sweet, honey
1763		(*E*)-2-Undecenal	Rancid
1767	1106	2-Acetyl-2-thiazoline	Popcorn, pork scratching
1778	1233	Methyl phenylacetate	Honey
1786		(*E, Z*)-2,6-Nonadienol	Rancid, toasted
1797		Citronellol	Citrus
1805	1329	Ethyl phenylacetate	Honey
1807	1360	2,4,6-Trichloroanisole	Cork, humidity
1811	1342	(*E, E*)-2,4-Decadienal	Fatty, aldehyde
1812	1254	2-Phenylethyl acetate	Roses
1820	1388	β-Damascenone	Boiled apple, sweet, compote
1825	1373	Geosmine	Mould, wet land, mustiness
1829	1308	(*E*)-Anethole	Anise
1860	1134	3-Mercaptohexanol	Thiol, green
1860	989	Hexanoic acid	Green, unpleasant
1862	1262	Geraniol	Floral, rose, citrus
1865	1100	Guaiacol	Medicinal, spiced
1879	1434	α-Ionone	Sweet, fruit, violet
1884	1064	Benzyl alcohol	Grass
1887	1370	Ethyl dihydrocinnamate	Sweet, floral
1913	1289	γ-Octalactone	Coconut
1944	1116	β-Phenylethanol	Roses
1957	1488	β-Ionone	Floral, violet, berry
1958	1134	(*Z*)-Whisky lactone	Coconut, cinnamon, wood
1976	1381	(*E*)-Whisky lactone	Coconut
2001		δ-Octalactone	Sweet
2015	1077	*o*-Cresol	Phenolic, medicinal
2032	1386	γ-Nonalactone	Sweet, peach
2034	1319	4-Ethylguaiacol	Clove, phenolic
2043	1302	Diethyl malate	Rose, sweet
2047	1092	Furaneol (4-hydroxy-2,5-dimethyl-3(2*H*)-furanone)	Candy, sweet, candyfloss
2074	1175	Homofuraneol	Candyfloss, peach
2077		Unknown	Rose, sweet, phenolic
2080	1190	Octanoic acid	Rancid, perspiration, plastic
2094	1103	*p*-Cresol	Animal, leather, stable
2100	1103	*m*-Cresol	Stable, animal, leather
2113		Tetrachloroanisol	Chlorine
2116	1404	4-Propylguaiacol	Clove, aromatic herbs
2125	1241	2,6-Dichlorophenol	Spiced, leaf
2135		Ethyl cinnamate	Floral, sweet
2155	1540	Bis(2-Methyl-3-furyl) disulphide	Fried, popcorn, toasted
2160	1250	2-Phenoxyethanol	Spiced, plasticine
2177		Eugenol	Clove, spiced, phenolic
2179	1486	γ-Decalactone	Spiced, wood phenolic
2184	1154	4-Ethylphenol	Phenolic, leather
2200	1154	3-Ethylphenol	Phenolic, leather
2210	1110	Sotolon	Liquorice, toasted, curry
2225		*o*-Aminoacetophenone	Muscatel, grape
2242	1328	4-Vinylguaiacol (2-methoxy-4-vinylphenol)	Clove, phenolic
2260	1490	Massoialactone	Coconut, fruit
2270	1586	γ-Undecalactone	Peach, sweet
2272		Decanoic acid	Rancid, perspiration
2285	1395	2,6-Dimethoxyphenol	Phenolic
2313		Unknown	Spiced, phenolic
2319		Tribromoanisole	Chlorine
2341	1462	δ-Undecalactone	Sweet
2375		2,4,6-Trichlorophenol	Medicinal, chlorine
2375	1465	Isoeugenol	Spice, mint, confectioner, sweet
2384	1686	γ-Dodecalactone	Peach, sweet
2433		2,3,6-Trichlorophenol	Medicinal, chlorine
2463		Indole	Tarmac, faeces
2465		Benzophenone	Boiled apple
2507		Skatole	Faeces
2535		2,4,5-Trichlorophenol	Medicinal, chlorine
2541		2,3,4- Trichlorophenol	Medicinal, chlorine
2570	1249	Phenylacetic acid	Honey, sweet
2592	1410	Vanillin	Vanilla, custard
2646		Methyl vanillate	Burnt, burnt vanilla
2665	1560	Ethyl vanillate	Vanilla
2683		Acetovanillone	Vanilla
3084		2,3,4,6-Tetrachlorophenol	Medicinal, chlorine

## References

[1] Saenz-Navajas M.P., Ballester J., Pecher C., Peyron D., Valentin D. (2013). Sensory drivers of intrinsic quality of red wines Effect of culture and level of expertise. Food Res. Int..

[2] Picard M., Tempere S., de Revel G., Marchand S. (2015). A sensory study of the ageing bouquet of red Bordeaux wines: A three-step approach for exploring a complex olfactory concept. Food Qual. Prefer..

[3] Parr W.V., Mouret M., Blackmore S., Pelquest-Hunt T., Urdapilleta I. (2011). Representation of complexity in wine: Influence of expertise. Food Qual. Prefer..

[4] Spence C., Wang Q.J. (2018). What does the term ’complexity’ mean in the world of wine?. Int. J. Gastron. Food Sci..

[5] Lytra G., Tempere S., Marchand S., de Revel G., Barbe J.C. (2016). How do esters and dimethyl sulphide concentrations affect fruity aroma perception of red wine? Demonstration by dynamic sensory profile evaluation. Food Chem..

[6] Wen Y., Lopez R., Ferreira V. (2018). An automated gas chromatographic-mass spectrometric method for the quantitative analysis of the odor-active molecules present in the vapors emanated from wine. J. Chromatogr. A.

[7] Loscos N., Hernandez-Orte P., Cacho J., Ferreira V. (2007). Release and formation of varietal aroma compounds during alcoholic fermentation from nonfloral grape odorless flavor precursors fractions. J. Agric. Food Chem..

[8] De-la-Fuente-Blanco A., Sáenz-Navajas M.-P., Valentin D., Ferreira V. (2020). Fourteen ethyl esters of wine can be replaced by simpler ester vectors without compromising quality but at the expense of increasing aroma concentration. Food Chem..

[9] De-la-Fuente-Blanco A., Sáenz-Navajas M.-P., Ferreira V. (2016). On the effects of higher alcohols on red wine aroma. Food Chem..

[10] Fuller G.H., Tisserand G.A., Steltenkamp R. (1964). Gas Chromatograph with human sensor—Perfumer model. Ann. N. Y. Acad. Sci..

[11] Wildenradt H.L., Christensen E.N., Stackler B., Caputi A., Slinkard K., Scutt K. (1975). Volatile constituents of grape leaves. I. Vitis Vinifera variety Chenin blanc. Am. J. Enol. Vitic..

[12] Stern D.J., Guadagni D., Stevens K.L. (1975). Aging of wine—Qualitative changes in volatiles of Zinfandel wine during 2 years. Am. J. Enol. Vitic..

[13] Rapp A., Knipser W., Engel L., Ullemeyer H., Heimann W. (1980). Off-falvor compounds in the berry and wine aroma of grapevine hybrids. I. The strawberry-like flavor. Vitis.

[14] Acree T., Barnard J., Cunningham D. (1984). A procedure for the sensory analysis of gas chromatographic effluents. Food Chem..

[15] Schieberle P., Grosch W. (1987). Evaluation of the flavor of wheat and rye bread crusts by Aroma Extract Dilution Analysis. Zeitschrift Lebensmittel-Untersuchung Forschung.

[16] Guth H. (1997). Identification of character impact odorants of different white wine varieties. J. Agric. Food Chem..

[17] Guth H. (1997). Quantitation and sensory studies of character impact odorants of different white wine varieties. J. Agric. Food Chem..

[18] Abbott N., Etievant P., Langlois D., Lesschaeve I., Issanchou S. (1993). Evaluation of the representativeness of the odor of beer extracts prior to analysis by GC eluate sniffing. J. Agric. Food Chem..

[19] Moio L., Chambellant E., Lesschaeve I., Issanchou S., Schlich P., Etievant P.X. (1995). Production of representative wine extracts for chemical and olfactory analysis. Ital. J. Food Sci..

[20] Priser C., Etievant P.X., Nicklaus S., Brun O. (1997). Representative champagne wine extracts for gas chromatography olfactometry analysis. J. Agric. Food Chem..

[21] Engel W., Bahr W., Schieberle P. (1999). Solvent assisted flavour evaporation—A new and versatile technique for the careful and direct isolation of aroma compounds from complex food matrices. Eur. Food Res. Technol..

[22] Ferreira V., Ortega L., Escudero A., Cacho J.F. (2000). A comparative study of the ability of different solvents and adsorbents to extract aroma compounds from alcoholic beverages. J. Chromatogr. Sci..

[23] Ferreira V., Jarauta I., Ortega L., Cacho J. (2004). Simple strategy for the optimization of solid-phase extraction procedures through the use of solid–liquid distribution coefficients: Application to the determination of aliphatic lactones in wine. J. Chromatogr. A.

[24] Cullere L., Bueno M., Cacho J., Ferreira V. (2010). Selectivity and efficiency of different reversed-phase and mixed-mode sorbents to preconcentrate and isolate aroma molecules. J. Chromatogr. A.

[25] Ferreira V., Jarauta I., Lopez R., Cacho J. (2003). Quantitative determination of sotolon, maltol and free furaneol in wine by solid-phase extraction and gas chromatography-ion-trap mass spectrometry. J. Chromatogr. A.

[26] Saenz-Navajas M.P., Campo E., Cullere L., Fernandez-Zurbano P., Valentin D., Ferreira V. (2010). Effects of the Nonvolatile Matrix on the Aroma Perception of Wine. J. Agric. Food Chem..

[27] Cullere L., Escudero A., Cacho J., Ferreira V. (2004). Gas Chromatography-Olfactometry and chemical quantitative study of the aroma of six premium quality Spanish aged red wines. J. Agric. Food Chem..

[28] Escudero A., Gogorza B., Melus M.A., Ortin N., Cacho J., Ferreira V. (2004). Characterization of the aroma of a wine from Maccabeo. Key role played by compounds with low odor activity values. J. Agric. Food Chem..

[29] Lyu J., Ma Y., Xu Y., Nie Y., Tang K. (2019). Characterization of the key aroma compounds in Marselan wine by Gas Chromatography-Olfactometry, quantitative measurements, aroma recombination, and omission tests. Molecules.

[30] Lan Y.-B., Xiang X.-F., Qian X., Wang J.-M., Ling M.-Q., Zhu B.-Q., Liu T., Sun L.-B., Shi Y., Reynolds A.G. (2019). Characterization and differentiation of key odor-active compounds of ’Beibinghong’ icewine and dry wine by gas chromatography-olfactometry and aroma reconstitution. Food Chem..

[31] Arthur C.L., Pawliszyn J. (1990). Solid-phase microextraction with thermal-desorption using fused-silica optical fibers. Anal. Chem..

[32] Ferreira V., Herrero P., Zapata J., Escudero A. (2015). Coping with matrix effects in headspace solid phase microextraction gas chromatography using multivariate calibration strategies. J. Chromatogr. A.

[33] Siebert T.E., Barter S.R., de Barros Lopes M.A., Herderich M.J., Francis I.L. (2018). Investigation of ’stone fruit’ aroma in Chardonnay, Viognier and botrytis Semillon wines. Food Chem..

[34] Ubeda C., Kania-Zelada I., del Barrio-Galán R., Medel-Marabolí M., Gil M., Peña-Neira Á. (2019). Study of the changes in volatile compounds, aroma and sensory attributes during the production process of sparkling wine by traditional method. Food Res. Int..

[35] Herrington J.S., Gomez-Rios G.A., Myers C., Stidsen G., Bell D.S. (2020). Hunting Molecules in Complex Matrices with SPME Arrows: A Review. Separations.

[36] Bensafi M., Pouliot S., Sobel N. (2005). Odorant-specific Patterns of Sniffing during Imagery Distinguish ’Bad ’ and ’Good’ Olfactory Imagers. Chem. Senses.

[37] Sobel N., Khan R.M., Hartley C.A., Sullivan E.V., Gabrieli J.D.E. (2000). Sniffing longer rather than stronger to maintain olfactory detection threshold. Chem. Senses.

[38] Escudero A., San-Juan F., Franco-Luesma E., Cacho J., Ferreira V. (2014). Is orthonasal olfaction an equilibrium driven process? Design and validation of a dynamic purge and trap system for the study of orthonasal wine aroma. Flavour Fragr. J..

[39] San-Juan F., Pet’ka J., Cacho J., Ferreira V., Escudero A. (2010). Producing headspace extracts for the gas chromatography-olfactometric evaluation of wine aroma. Food Chem..

[40] Lopez P., Batlle R., Nerin C., Cacho J., Ferreira V. (2007). Use of new generation poly(styrene-divinylbenzene) resins for gas-phase trapping-thermal desorption—Application to the retention of seven volatile organic compounds. J. Chromatogr. A.

[41] Luong J., Gras R., Jennings W. (2007). An advanced solventless column test for capillary GC columns. J. Sep. Sci..

[42] Wang J.M., Gambetta J.M., Jeffery D.W. (2016). Comprehensive study of volatile compounds in two Australian Rose wines: Aroma Extract Dilution Analysis (AEDA) of extracts prepared using Solvent-Assisted Flavor Evaporation (SAFE) or Headspace Solid-Phase Extraction (HS-SPE). J. Agric. Food Chem..

[43] Van Ruth S.M. (2001). Methods for gas chromatography-olfactometry: A review. Biomol. Eng..

[44] Plutowska B., Wardencki W. (2008). Application of gas chromatography-olfactometry (GC-O) in analysis and quality assessment of alcoholic beverages—A review. Food Chem..

[45] Lawless H.T., Heymann H. (2010). Sensory Evaluation of Food: Principles and Practices.

[46] Pollien P., Ott A., Montigon F., Baumgartner M., MunozBox R., Chaintreau A. (1997). Hyphenated headspace gas chromatography sniffing technique: Screening of impact odorants and quantitative aromagram comparisons. J. Agric. Food Chem..

[47] Van Ruth S.M., Roozen J.P. (1994). Gas-Chromatography sniffing port analysis and sensory evaluation of commercially dried bell peppers (capsicum-annuum) after rehydration. Food Chem..

[48] Delahunty C.M., Eyres G., Dufour J.P. (2006). Gas chromatography-olfactometry. J. Sep. Sci..

[49] Pollien P., Fay L.B., Baumgartner M., Chaintreau A. (1999). First attempt of odorant quantitation using Gas Chromatography−Olfactometry. Anal. Chem..

[50] McDaniel M.R., Miranda-Lopez R., Watson B.T., Micheals N.J., Libbey L.M. (1990). Pinot noir aroma: A sensory/gas chromatographic approach. Dev. Food Sci..

[51] Etiévant P.X., Callement G., Langlois D., Issanchou S., Coquibus N. (1999). Odor intensity evaluation in Gas Chromatography—Olfactometry by Finger Span method. J. Agric. Food Chem..

[52] Stevens S. (1975). Cross-modality matching. Psychophysics.

[53] Stevens S., Stone G. (1959). Finger span: Ratio scale, category scale, and JND scale. J. Exp. Psychol..

[54] Pet’ka J., Ferreira V., Cacho J. (2005). Posterior evaluation of odour intensity in gas chromatography-olfactometry: Comparison of methods for calculation of panel intensity and their consequences. Flavour Fragr. J..

[55] Dravnieks A. (1985). Atlas of Odor Character Profiles.

[56] Ferreira V., Pet’ka J., Aznar M., Cacho J. (2003). Quantitative gas chromatography-olfactometry. Analytical characteristics of a panel of judges using a simple quantitative scale as gas chromatography detector. J. Chromatogr. A.

[57] Ferreira V., Pet’ka J., Aznar M. (2002). Aroma Extract Dilution Analysis. Precision and optimal experimental design. J. Agric Food Chem..

[58] Ferreira V., Ortin N., Escudero A., Lopez R., Cacho J. (2002). Chemical characterization of the aroma of Grenache rose wines: Aroma extract dilution analysis, quantitative determination, and sensory reconstitution studies. J. Agric. Food Chem..

[59] Frijters J.E.R. (1978). Critical analysis of odor unit number and its use. Chem. Senses Flavour.

[60] Stevens S.S. (1961). To honor Fechner and repeal his law—A power function, not a LOG function, describes operating characteristics of a sensory system. Science.

[61] Etiévant P.X., Jackson J.F., Linskens H.F. (2002). Odour intensity evaluation in GC-Olfactometry by Finger Span method. Analysis of Taste and Aroma.

[62] Campo E., Ferreira V., Escudero A., Cacho J. (2005). Prediction of the wine sensory properties related to grape variety from dynamic-headspace gas chromatography-olfactometry data. J. Agric Food Chem..

[63] Campo E., Cacho J., Ferreira V. (2008). The chemical characterization of the aroma of dessert and sparkling white wines (Pedro Ximenez, Fino, Sauternes, and Cava) by gas chromatography-olfactometry and chemical quantitative analysis. J. Agric Food Chem..

[64] Saenz-Navajas M.P., Alegre Y., de-la-Fuente A., Ferreira V., Garcia D., Eizaguirre S., Razquin I., Hernandez-Orte P. (2016). Rapid sensory-directed methodology for the selection of high-quality aroma wines. J. Sci. Food Agric..

[65] Alegre Y., Saenz-Navajas M.P., Ferreira V., Garcia D., Razquin I., Hernandez-Orte P. (2017). Rapid strategies for the determination of sensory and chemical differences between a wealth of similar wines. Eur. Food Res. Technol..

[66] Barata A., Campo E., Malfeito-Ferreira M., Loureiro V., Cacho J., Ferreira V. (2011). Analytical and sensorial characterization of the aroma of wines produced with sour rotten grapes using GC-O and GC-MS: Identification of key aroma compounds. J. Agric. Food Chem..

[67] Ferreira V., San Juan F., Escudero A., Cullere L., Fernandez-Zurbano P., Saenz-Navajas M.P., Cacho J. (2009). Modeling quality of premium Spanish red wines from Gas Chromatography-Olfactometry data. J. Agric. Food Chem..

[68] Ferreira V., Moreno-Arribas M.V., Polo M.C. (2009). Identification of impact odorants of wines. Wine Chemistry and Biochemistry.

[69] Grob K., Grob G. (1981). Testing capillary Gas-Chromatographic columns. J. Chromatogr..

[70] Campo E., Ferreira V., Lopez R., Escudero A., Cacho J. (2006). Identification of three novel compounds in wine by means of a laboratory-constructed multidimensional gas chromatographic system. J. Chromatogr. A.

[71] Gerstel. https://www.gerstel.com/en/olfactory-detection-port.htm.

[72] Pubchem. https://pubchem.ncbi.nlm.nih.gov/.

[73] Pherobase. https://www.pherobase.com/kovats/.

[74] Flavornet. https://www.flavornet.org/f_kovats.html.

[75] Molyneux R.J., Schieberle P. (2007). Compound identification: A Journal of agricultural and food chemistry perspective. J. Agric. Food Chem..

[76] Molyneux R.J., Beck J.J., Colegate S.M., Edgar J.A., Gaffield W., Gilbert J., Hofmann T., McConnell L.L., Schieberle P. (2019). Guidelines for unequivocal structural identification of compounds with biological activity of significance in food chemistry (IUPAC Technical Report). Pure Appl. Chem..

